# Comparison of *Schistosoma mansoni* Soluble Cercarial Antigens and Soluble Egg Antigens for Serodiagnosing Schistosome Infections

**DOI:** 10.1371/journal.pntd.0001815

**Published:** 2012-09-13

**Authors:** Huw Smith, Mike Doenhoff, Cara Aitken, Wendi Bailey, Minjun Ji, Emily Dawson, Henk Gilis, Grant Spence, Claire Alexander, Tom van Gool

**Affiliations:** 1 Scottish Parasite Diagnostic Reference Laboratory, Department of Bacteriology, Stobhill Hospital, Glasgow, Scotland; 2 School of Biology, University of Nottingham, University Park, Nottingham, United Kingdom; 3 Diagnostic Parasitology Laboratory, Liverpool School of Tropical Medicine, Liverpool, United Kingdom; 4 Department of Pathogen Biology, Nanjing Medical University, Nanjing, China; 5 Parasitology Section, Department of Medical Microbiology, Academic Medical Center, Amsterdam, The Netherlands; University of Queensland, Australia

## Abstract

A *Schistosoma mansoni* cercarial antigen preparation (cercarial transformation fluid – SmCTF) was evaluated for detection of anti-schistosome antibodies in human sera in 4 collaborating laboratories. The performance of SmCTF was compared with that of *S. mansoni* egg antigens (SmSEA) in an indirect enzyme-immunoassay (ELISA) antigen assay, the latter being used routinely in 3 of the 4 participating laboratories to diagnose *S. mansoni* and *S. haematobium* infections. In the fourth laboratory the performance of SmCTF was compared with that of *S. japonicum* egg antigens (SjSEA) in ELISA for detection of anti-*S. japonicum* antibodies. In all 4 laboratories the results given by SmCTF in ELISA were very similar to those given by the antigen preparation routinely used in the respective laboratory to detect anti-schistosome antibodies in human infection sera. In so far as the ELISA results from SmCTF are thus so little different from those given by schistosome egg antigens and also cheaper to produce, the former is a potentially useful new diagnostic aid for schistosomiasis.

## Author Summary

Diagnosis of schistosomiasis is problematic since no method is yet available that gives both 100% sensitivity and 100% specificity. The method traditionally used is microscopy, but because of inherent insensitivity this technique often wrongly diagnoses patients as uninfected. Use of serological assays involving detection of specific antibodies is now increasing since the putative sensitivity of these tests is much higher than that of other alternative methods of diagnosis. They are routinely used in travellers' medicine clinics where often only light infections are encountered which microscopy is not sensitive enough to detect. ELISA incorporating schistosome soluble egg antigens (SEA) is often the antibody-detection test of choice. The use of the SEA-ELISA for diagnosis of schistosomiasis in developing countries is however restricted since SEA is relatively expensive to produce. Here we investigated whether a cheaper alternative *S. mansoni* antigenic preparation derived from schistosome cercariae (SmCTF) could potentially replace SEA in ELISA. Our results demonstrate that SmCTF performs equivalently to *S. mansoni* SEA for the diagnosis of both *S. mansoni* and *S. haematobium* infections, and that SmCTF is also as good as *S. japonicum* SEA for the diagnosis of schistosomiasis japonica. We discuss how even more affordable and practical diagnostic aids for schistosomiasis might be developed.

## Introduction

More than 200 million people in over 70 countries world-wide are infected with schistosomes with infection-induced morbidity being particularly pronounced in sub-Saharan Africa [1], [2]. Humans become infected as a result of swimming, bathing and fishing in water in which infected intermediate host snails have released free-swimming cercariae that can penetrate human skin.

The heaviest schistosome infections are generally found in children and young adults and in recognition of this school children are the main target of schistosomiasis control programmes based on treatment with praziquantel. Prior to instigating control the prevalence and intensity of infection is generally estimated by microscopic detection of eggs in faecal or urine samples, which is a relatively slow and laborious process.

Insensitivity is another serious defect of egg detection methods of diagnosis, especially of the intestinal schistosome infections [3], [4] and many light infections are missed because of the absence of eggs in the small volumes of excreta that can be routinely examined microscopically [5]–[9] These limitations impose significant constraints on current control initiatives [10], [11] Considerable effort has been expended in the effort to develop immunodiagnostic tests that are an improvement on microscopical parasitology. It has been argued that methods to detect circulating or excreted schistosome antigens are desirable because they are likely to reflect active infection most accurately. However, the sensitivity of antigen detection tests seems to be no better than that of microscopy, particularly with regard to detection of faecally-excreted eggs of *S. mansoni* and in situations in which low egg counts pertain [12], [13]

Antibody detection tests have often been deemed unsuitable for diagnosis of schistosomiasis, mainly because of their apparent lack of specificity and inability to distinguish active from inactive infection – namely the common observation that many subjects that are antibody-positive are egg-negative by microscopy. However, possible alternative explanations for the lack of specificity are that the many instances of antibody-positivity, egg-negativity reflect the failure of insensitive microscopy to detect eggs in subjects who are lightly-infected [3] or who have been treated with sub-curative drug doses [14]. Indeed it has been demonstrated that in some patients antibody levels do decline following treatment [15], particularly antibodies against the soluble egg antigen fraction CEF6 and patients with more steeply declining anti-CEF6 antibody titres were considered to have been better cured than those with titres that remained higher [16]. There is of course also the possibility that antibody false-positives are due to heterologous infectious agents. Despite their failings, antibody-detection is for some time likely to remain the best available method for diagnosis in areas of low intensity of schistosome infection [11], [17].Tourists and other visitors to schistosome endemic areas who become infected with schistosomes commonly only have light infections and because praziquantel is such a safe drug travellers' medicine clinics now often base their treatment decisions on the result of an antibody-detection diagnostic test alone. Soluble *S. mansoni* egg antigens (SmSEA) in enzyme immunosorbant assay (ELISA) formats are frequently the diagnostic method of choice in these clinics [18]–[20] and such immunoassays can also give meaningful results in endemic areas [21].

SmSEA is however a relatively expensive commodity, requiring for its production the infection of relatively large numbers of laboratory animals (generally mice or hamsters), from the tissues of which the eggs are isolated after the infections become patent. Diagnostic tests based on SmSEA would therefore not be ideal for deployment in tropical countries with endemic schistosomiasis, most of which are resource-poor. Based on the evidence that much of the antibody induced by *S. mansoni* infections is specific for glycanic epitopes [22], [23] and that *S. mansoni* egg and cercarial molecules have many glycanic epitopes in common [24]–[26], we have begun to investigate whether soluble *S. mansoni* cercarial antigens (in the form of a preparation we have named ‘cercarial transformation fluid’ – SmCTF) can be substituted for SmSEA as an antibody target in ELISA. A preliminary study has indicated that SmCTF did indeed give very similar reactivity to SmSEA when reacted in ELISA with sera from schistosome-infected patients who had attended a travellers' medicine clinic in London [27]. In that study the sera, when tested previously, all had reacted positively with SmSEA, but they were designated just as schistosome-infected i.e., there was no differentiation between *S. mansoni* and *S. haematobium* infection. There was no difference between the rate of cross-reactivity of SmCTF with sera from patients with other infections than that of schistosomiasis compared to that achieved with SmSEA [27]. A second study has also demonstrated that SmCTF performs equivalently to SmSEA for diagnosis of *S. mansoni* infections in an endemic setting, and indicates that SmCTF may actually be more specific than SmSEA for diagnosis in endemic areas [28].In this study we have extended that work in a collaborative study involving 3 different parasite diagnosis laboratories in Europe: respectively, in Scotland, England and The Netherlands, all of which deal with travellers and immigrants from Africa or the Middle East who may be infected with *S. mansoni* and/or *S. haematobium*. The species of schistosome infecting many of the serum donors could be determined by prior clinical investigations routinely carried out in the respective laboratories, including travel history, water exposure questionnaire and/or microscopy of urine and stools for schistosome ova. The opportunity has also been taken to test the reactivity of human sera from an area endemic for *S. japonicum* in The People's Republic of China.

In all 4 settings the performance of SmCTF was compared directly with SmSEA in ELISA. In each of the four laboratories the ‘in-house’ procedures in routine use for performing ELISA were used to compare the antigen preparations, with therefore no effort having been made to co-ordinate or validate the methodology at this stage.

## Materials and Methods

### Ethics statement

All the serum samples used in this study were routine diagnostic samples which had previously been sent to the respective laboratory from other hospitals/medical practitioners with the request they be tested for anti-schistosome antibodies. The samples were therefore not collected specifically for this study and were taken from stored collections of sera.

In the case of the participating laboratory having collected the sample for a previous routine diagnosis, sample donors were asked if they had any objections to usage of the samples for future research. If no objection was provided, the samples were deemed suitable for use in this study: i.e., a determination of the merits of different diagnostic tests.

After processing every sample sent to one of the laboratories for testing was stored frozen in accordance with Standard Operating Procedures for “sample collection and storage” that are in place in each of the laboratories. All samples are maintained for a set period of time in case a sample requires retesting. The collections of sera so accrued provide reference material for research/development studies such as this.

The *S. japonicum* infection sera used by Nanjing Medical University (see below) had been collected as part of the National Key Technologies R&D Program for China Tenth Five-Year Plan (grant no. 2004BA718B05). The project was reviewed and approved by the institutional review board of Nanjing Medical University. The other 3 serum collections used in this study had been given in-house names which were and are used in references to them; e.g. SPDRL Sera or Amsterdam Medical Centre Serum Bank. These were in turn parts of larger reference collections: Scottish Parasite Clinical Reference Collection, the AMC Parasitology Serum Bank and the LSTM Diagnostic Parasitology Laboratory Serum Bank.

The regulations to which the participating laboratories adhered with regard to use of samples in their possession covered the use of these samples in this particular study. Application for IRB approval was deemed unnecessary and irrelevant because any sample which was received by any of the participating laboratories from elsewhere was given a unique identifier. No personal identifying data, such as name, date of birth, gender, and/or age were retained on the collection tube. All samples were stored in numerical order using a unique identification number to ensure they were anonymised for any subsequent study. Each sample used in this study was thus completely anonymised and the results were a) not entered onto any computerised reporting system; b) not accessible by any outside body, and c) not reported to the donor or the sample sender and therefore not reported to the patient.

In the participating laboratories it is standard practice for stored fully-anonymized serum samples to be used for evaluation of new diagnostic tests, as in this study, without the need for permission to be sought from regulatory bodies. Consent was not required for this particular study because it was simply a comparison of diagnostic methodologies using fully anonymised samples which were already part of collections of reference material. Each sample was being tested for the same parasitic infection for which it had initially been screened. No individual's results from this study have been reported elsewhere and no follow-up samples were necessary.

The work on laboratory animals was approved by the Ethical Review Procedure of the University of Nottingham. Further details are available at this link: http://www.nottingham.ac.uk/animalresearch/erp/erp.aspx. The work was conducted according to the UK Animal (Scientific Procedures) act 1986 with personal and project licence authorities held by MD (numbers PIL70/3255 and PPL40/3024 respectively). The study was conducted in accordance to Nottingham University's guidelines for animal husbandry which meet the UK Home Office Code of Practice for the Housing and Care of Animals used in Scientific Procedures. Further details are available at this link: http://www.nottingham.ac.uk/animalresearch/animalwelfare/animalwelfare.aspx.

### Collaborating laboratories

Four medical parasitology laboratories collaborated in this study to compare the performance of two *S. mansoni* antigen preparations in ELISA with sera from schistosome-infected people: The Scottish Parasite Diagnostic Reference Laboratory, Glasgow, Scotland (SPDRL), The Diagnostic Parasitology Laboratory at the Liverpool School of Tropical Medicine (LSTM), the Parasitology Section, Academic Medical Center, Netherlands (AMC) and the Department of Pathogen Biology, Nanjing Medical University, Nanjing, China (NJMU).

### Antigens

Two *S. mansoni* antigen preparations, SmCTF and SmSEA, were produced by BioGlab Ltd (Nottingham, UK) and distributed to the above-named 4 laboratories for comparison of their performance in ELISA.

SmCTF was prepared from cercariae, the shedding of which was induced from *Biomphalaria glabrata* snails. The cercariae were shed into deionized water at 30°C under a 60 W tungsten light and the larval suspensions concentrated by suction over a glass fibre filter, followed by gravity-sedimentation in the dark in approximately 20 ml water at 4°C. Excess water was removed from over the sedimented cercarial pellets, the latter being generally of 1 ml volume or more. The pellets were resuspended in 5 ml phosphate buffered saline (PBS, pH 7.2) and aspirated 15 times through a 20 gauge disposable syringe needle in 5 ml phosphate-buffered saline (pH 7.2) using a 5 ml disposable plastic syringe [29]. Suspensions of dispersed, ‘transformed’ schistosomula and their now separate tails were incubated in plastic Petri dishes for 45 min at room temperature (RT). After incubation schistosomula and tails were removed by centrifugation (5 min, 250× g) and the supernatant (SmCTF) was collected and stored at −80°C. The protein concentration of SmCTF was approximately 1.0 mg per ml; batches were aliquoted in 1 ml volumes, freeze-dried and distributed to the 4 collaborating laboratories for use in this study.

Extracts of *S. mansoni* soluble egg antigens (SmSEA) were prepared as described earlier [30].

*S. japonicum* egg antigens (SjSEA) were prepared with eggs taken from rabbits 45 days after they had been infected with approximately 1,000 *S. japonicum* cercariae. Livers were removed and kept at 4°C overnight in order to better isolate eggs. Eggs were harvested from the homogenized livers by differential centrifugation. The absence of contaminating rabbit tissue fragments in the egg preparation was checked by microscopic analysis. Purified eggs were suspended in PBS and homogenized on ice. After repeated freezing and thawing, the homogenate was centrifuged at 13,800 g at 4°C for 20 min, and the supernatant was used as *S. japonicum* soluble egg antigen (SjSEA).

### Antisera and enzyme-immunoassay (ELISA)

#### SPDRL

Antisera from 59 Officers Training Core (OTC) pupils who visited Uganda in 2006 and swam in the White Nile were tested. The donors were presumed to be potentially infected with *S. mansoni* by virtue of this species of schistosome being endemic in that area [31].

Antisera from 3 parasitologically-confirmed *S. haematobium* travellers were also tested, as were samples from 8 Glasgow schoolchildren who had visited Malawi for a period of 4 weeks in 2007 and who had swum in Lake Malawi, a known focus of endemic *S. haematobium* [32]. The latter were thus deemed to be potentially infected with *S. haematobium*.

For ELISA the wells of polystyrene, flat bottomed, duostrip microtitre strips (Greiner; www.gbo.org) were filled with 50 µl of carbonate-bicarbonate coating buffer (pH 9.6) containing either 2.5 µg of SmSEA protein or 2 µg SmCTF protein in 5 ml of coating buffer, and incubated in a humidified chamber at 37°C overnight. Excess antigens were removed by washing the wells 3 times with 250 µl of 150 mM phosphate buffered saline, pH 7.2 containing 0.05% (v/v) Tween 20 (PBS-T). Fifty µl aliquots of human serum, diluted to 1∶300 in 2.5% dried skimmed milk in PBS-T (pH 7.2) were incubated in the coated wells at room temperature (RT) for 30 min on a mechanical plate shaker, followed by 3 washes with PBS-T. Fifty µl of horseradish peroxidase-conjugated anti-human IgG (HRP-IgG; Sigma, Poole, UK) diluted 1∶900 in PBS-T were added to each well, followed by incubation for 1 h at 37°C and washing 3 times with PBS-T. Finally, the enzyme-substrate reaction was developed by the addition of 50 µl of freshly prepared TMB substrate solution (0.1 mg/ml 2, 2-azino-bis [3-ethylbenz-thiazoline-6-sulfonic acid] containing 25 µl 30% H_2_O_2_/100 ml) to each well. The reaction was developed for 30 min at RT and stopped by adding 50 µl of 0.18 M sulphuric acid into each well. The optical density (OD) of each well was measured at 450 nm in a Titertek microplate reader (Flow Laboratories, Herts., UK).

Chequer-board titrations were performed prior to analysis of test human sera to establish the amount of each antigen to coat the microtitre wells and the dilutions of the test sera and anti-human IgG-peroxidase conjugate. Based on four replicates of the 50 European negative samples tested, the cut off OD_450_ for positive sera, plus two standard deviations, was 0.22 for SmSEA and 0.25 for SmCTF. All antisera were tested in duplicate against both SmSEA and SmCTF.

#### LSTM

Patients suspected of having schistosomiasis submitted faecal and urine samples to the LSTM diagnostic lab. If eggs were not found in a random urine sample patients were asked to submit a 10am–2pm sample for membrane filtration. Faecal samples submitted for parasites were examined using a modified formol-ether concentration, up to 3 stools/patient being tested routinely.

A total of 88 sera were examined: antisera from 40 cases of parasitologically confirmed *S. haematobium* patients, seen as out-patients at LSTM between 1995 and 2009, were tested. 18 were travellers to Malawi and the remainder were from patients who had lived/worked in various African countries.

The 12 antisera from parasitologically confirmed *S. mansoni* patients were again referrals to LSTM, 3 were from a group who had travelled to Namibia, 2 were co-travellers to Ethiopia and the remainder had history of travel to Uganda and other African countries.

Thirty six sera from “suspected” cases of schistosomiasis were also tested. Patients in whom eggs had not been detected consisted of symptomatic patients with a history of Malawi travel (13), those with a history suggestive of exposure (swimming in African lakes), some with blood in urine, 2 with Katayama and others presenting with an eosiniphilia (but negative for strongyloides/filarial antibodies). A few samples were from members of expeditions in which other egg-positive individuals had already been found.

For ELISA flat-bottomed microtitre plates (ICN/Flow) were coated overnight with 100 µl antigen solution (1 µg protein/ml SmSEA or 0.8 µg protein/ml SmCTF) diluted in carbonate/bicarbonate coating buffer, pH 9.6. Excess antigens were removed by washing the wells 3× with 250 µl of 150 mM phosphate buffered saline, pH 7.2 containing 0.05% (v/v) Tween 20 (PBS-T). 100 µl of each serum diluted 1/200 in PBS+1% BSA+0.1%Tween was added to antigen-coated wells and incubated for 1 h at RT. After each step each well was subjected to 3×4 min washes with washing buffer (isotonic saline +0.5% Tween) until the substrate was added. After washing 100 µl alkaline phosphatase-conjugated anti-human IgG (Sigma) diluted 1∶3,500 in incubation buffer (PBS/Tween) was added for 1 hour. 100 µl liquid pNPP substrate (Southern Biotech) was added and monitored until the OD of negative controls reached a pre-determined OD (0.080–0.110) and positive controls an OD >0.800 (around 30–40 minutes). ODs were read at 405 nm on a BioRad iMark microplate reader. The cut-off point was determined using 52 sera from control subjects with no history of exposure to schistosomiasis. This was determined to be 0.200 for both CTF and SEA antigens.

#### AMC

Antisera: a total of 41 sera were tested. The sera were collected within 1 month of parasitological and clinical examination and stored at −20°C until use. Twenty one sera were from patients with parasitologically proven *Schistosoma* infection: 10 from patients with *S. mansoni* infection; 5 patients with *S. haematobium* infection; and 6 patients with Katayama fever. A further 20 sera that were positive in routine serology in the AMC hospital were used: 9 patients positive in both IHA (Fumouze) and SEA-ELISA; 6 patients that were IHA negative, SEA-ELISA positive; and 5 patients that were IHA positive and SEA-ELISA negative. Five sera were used as negative controls, having been found negative after routine serological testing for presence of anti-schistosome antibodies.

For ELISA, wells of flat-bottomed polystyrene high-bind microplates (Corning, Inc., Acton, MA) were coated overnight at 4°C with 30 ul volumes of SmCTF (1.3 µg protein/ml) or SmSEA (1.3 µg protein/ml) in 50 mM carbonate/bicarbonate buffer (pH 9.6). Subsequently, the wells were treated with 30 µl PBS, pH 7.2, containing 0.05% Tween 20 and 1% egg albumin (PBTWEG) for 30 min at 37°C. After washing three times with PBS containing 0.05% Tween 20 (PBTW) 30 µl of each serum sample (diluted 1∶400 in PBTWEG) were added and incubated for 1 h at 37°C. After washing five times with PBTW 30 µl horseradish peroxidase-conjugated goat anti-human IgG (Nordic) diluted 1∶1500 in PBTWEG was added for 30 min at 37°C. The wells were washed five times with PBTW, and 30 µl substrate solution (0.1% 5-amino salicylic acid in phosphate buffer, pH 5.95, with 0.03% H_2_O_2_; Merck, Whitehouse Station, NJ) was added for 1 h in the dark at RT. The optical density (OD) was read at 492 nm on a Multiskan Ascent reader (Labsystems, Helsinki, Finland). The cut-off OD_492_ values for both SmSEA and SmCTF was 0.22.

#### NMU

Sera from 42 people were collected in Jiahu village located on the Southeastern shore of Poyang Lake, Jiangxi Province, China. *S. japonicum* infection was confirmed in 25 of the donors by detection of *S. japonicum* eggs in the faeces. The remaining 17 subjects were egg-negative. Twenty sera from volunteers without a history of residence in a schistosome- endemic area were used as negative controls. All sera were stored at −70°C until use.

For ELISA chequerboard titrations were performed to determine optimum antigen concentrations of SjSEA, SmSEA and SmCTF, and the dilutions of human sera and horse radish peroxidise(HRP)-conjugated goat anti-human IgG.

Flat-bottomed microwell plates (Costar, Corning Incorporated Corning, New York, NY, USA) were coated overnight at 4°C with 100 µl SjSEA, SmSEA or SmCTF (all three antigens being at a concentration of 0.2 µg protein/ml) and then washed. After adding 200 µl blocking buffer (5% skimmed milk) in PBS (pH7.2) the plates were incubated at 37°C for 1 h at RT and then washed. All test sera were diluted 1∶250 with PBS, and 100 µl was added to each well. The plates were incubated at 37°C for 2 h and then washed. HRP-conjugated goat anti-human IgG (Serotec) was diluted 1∶6000 with PBS, and 100 µl was added to each well and incubated at 37°C for 1 h before the plates were washed. The plates were developed with TMB substrate solution (0.2 mg/ml 2,2-azino-bis [3-ethylbenz-thiazoline-6-sulfonic acid] containing 50 µl 30% H_2_O_2_/100 ml) and the reaction stopped by adding 100 µl H_2_SO_4_ (2 M) after 15 min. The plates were read at 450 nm, using an ELISA reader (Bio-Rad mod. 550). Each serum sample was tested in duplicate.

### Statistical analysis

All data were analysed using GraphPad Prism 4 and GraphPad InStat 3 (GraphPad Software, USA). Correlations were calculated using either Spearman's r correlation or Pearson's r correlation dependent upon the normality of the data. Sensitivities and specificities were calculated using the SmSEA-ELISA as the ‘gold-standard’ test unless elsewhere stated. Statistical differences in mean SmSEA OD readings and SmCTF OD readings were calculated using a t-test or the Wilcoxon matched pairs test as appropriate. Statistical differences between mean OD readings of one species compared to another were calculated using either Welch corrected unpaired t-tests or Mann Whitney U tests. Significance was assigned at p<0.05.

## Results

### SPDRL

**Figure 1:**
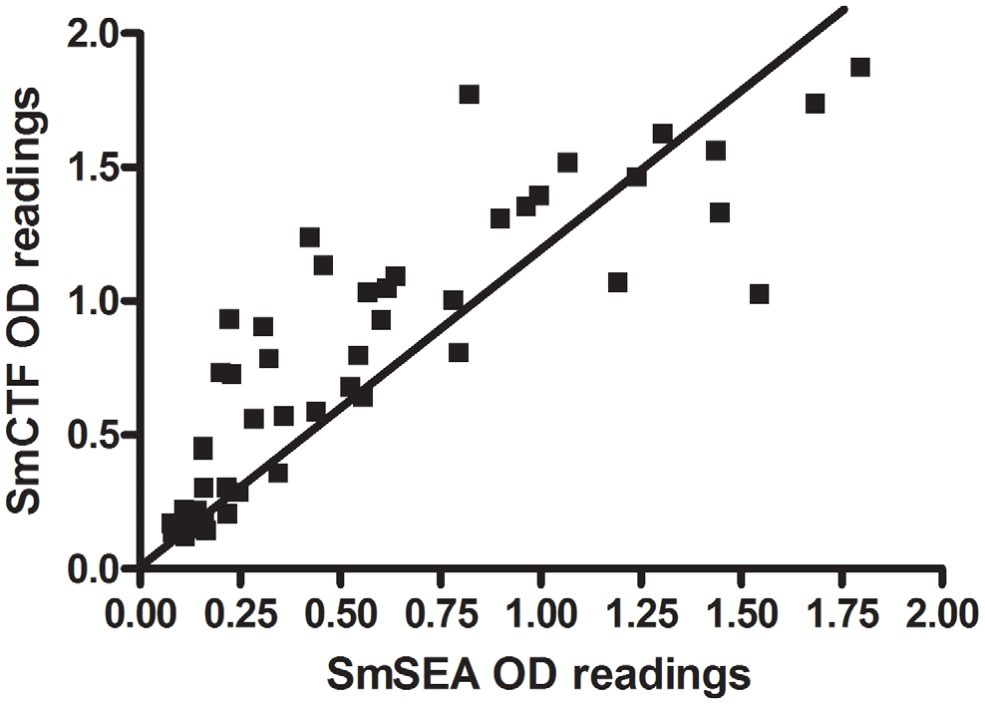
SPDRL: correlation between *S. mansoni* cercarial transformation fluid (SmCTF) and soluble egg antigen (SmSEA) in ELISA. Results for sera from suspected *S. mansoni*-infected patients (59 school pupils who attended a training course in the vicinity of the White Nile in Uganda).

Figure 1 shows the correlation between respective anti-SmCTF and anti-SmSEA OD_450_ readings from sera of 59 school pupils who attended a training course in the vicinity of the White Nile in Uganda. There was 91% correlation (95% CI 85.1–94.6; p<0.0001). It is apparent that the majority of individual anti-SmCTF OD_450_ readings (mean = 0.71, SD = 0.53) are greater than the respective OD_450_ anti-SmSEA OD_450_ values (mean = 0.50, SD = 0.47); a difference which is significant (p<0.0001). The sensitivity of the SmCTF compared with the SmSEA was 97.1% and the specificity was 80%. The positive predictive value was 86.8% and the negative predictive value was 95.3%.

**Figure 2:**
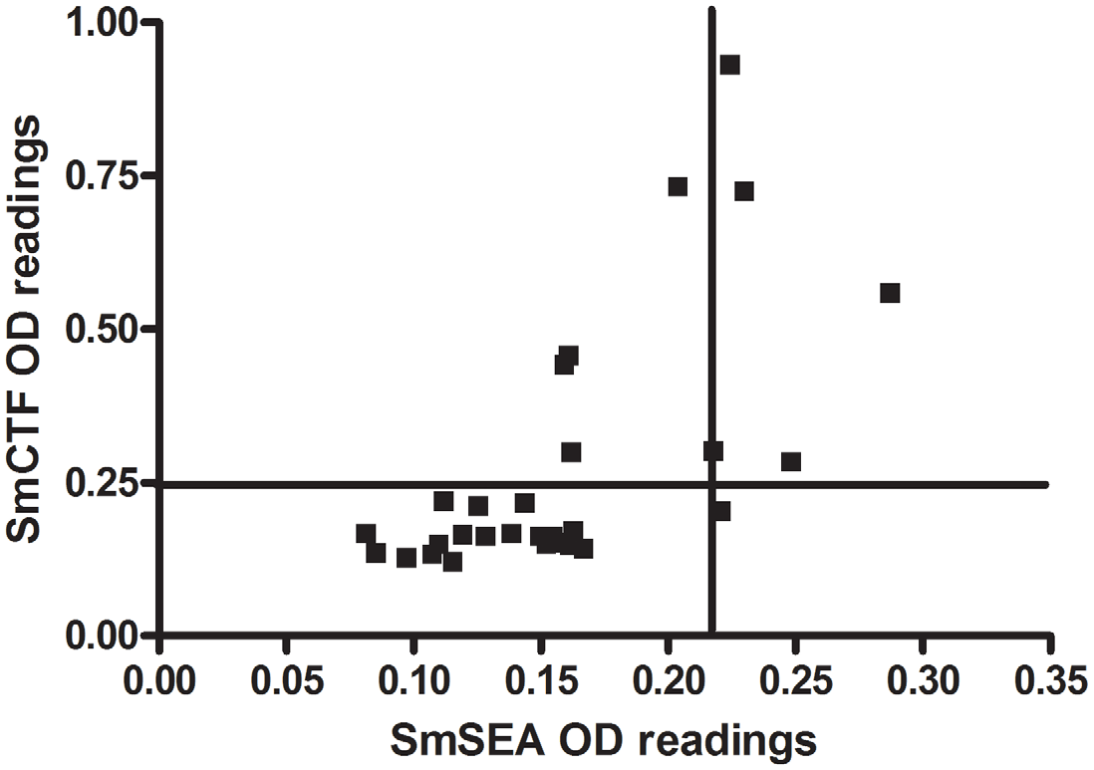
SPDRL: correlation between *S. mansoni* cercarial transformation fluid (SmCTF) and soluble egg antigen (SmSEA) in ELISA. Results for sera from those subjects in Figure 1 that had anti-SmSEA OD450 values of between 0 and 0.3. Vertical and horizontal lines indicate the cut-off values of, respectively, 0.22 for SmSEA and 0.25 for SmCTF.

Figure 2 shows the anti-SmCTF and anti-SmSEA OD_450_ readings for those of the 59 subjects in Figure 1 who had anti-SmSEA ODs of between 0 and 0.3. The cut-off values for SmCTF and SmSEA, estimated from the ELISA results of 50 negative controls, were respectively 0.25 for SmCTF and 0.22 for SmSEA and have been indicated in the graph. Five sera gave ODs which were above the SmCTF cut-off, but below the SmSEA cut-off; i.e., recorded a positive result with the former antigen, negative with the latter, a result consistent with SmCTF being more reactive than SmSEA with *S. mansoni* infection sera in this laboratory.

**Figure 3:**
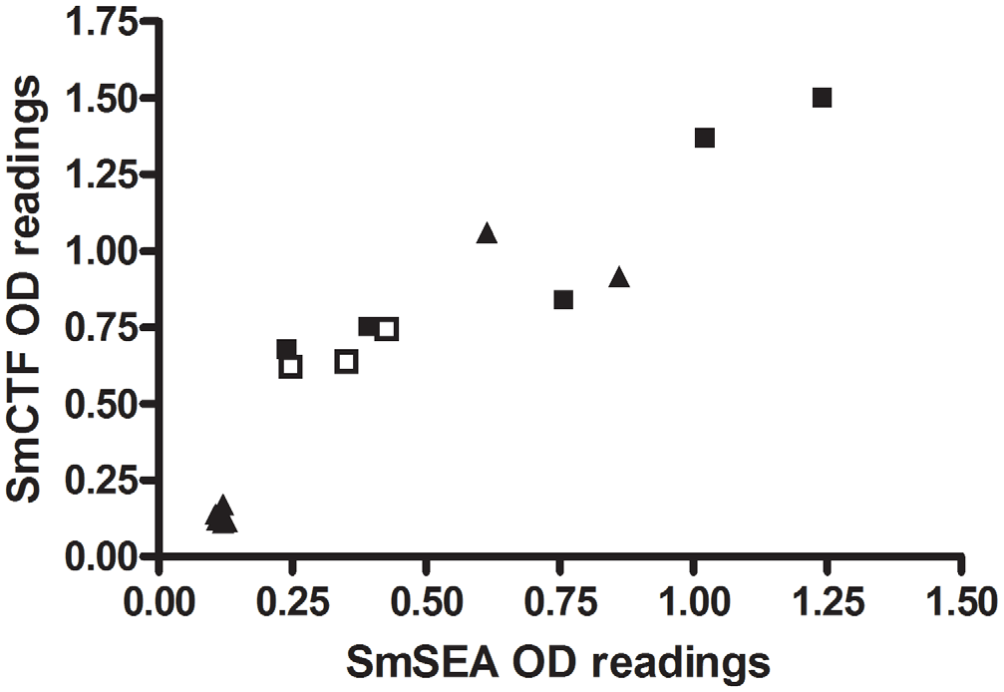
SPDRL: correlation between *S. mansoni* cercarial transformation fluid (SmCTF) and soluble egg antigen (SmSEA) in ELISA. Results for sera from 8 subjects previously shown parasitologically to be infected with *S. haematobium* and 8 subjects suspected to be infected with *S. haematobium*. ▴ = 2 positive and 6 negative sera from patients that had visited Lake Malawi; □ = patients with *S. haematobium* eggs in urine; ▪ = control positive sera from patients known to be *S. haematobium*-infected.

Figure 3 shows individual SmCTF OD_450_ values plotted against the respective SmSEA OD_450_ values of sera from 8 subjects known to be infected with *S. haematobium* by virtue of the presence of eggs in urine and also from 8 school children who had visited Lake Malawi, and thereby assumed also to have been exposed to *S. haematobium*. Two of the 8 children were positive for both antigens and 6 were negative, the latter showing as a closely knit group in the lower left corner of the figure. There was 90.8% correlation between the SmCTF OD_450_ values and the SmSEA OD_450_ values (95% CI 74.3–96.9; p<0.0001), and no significant difference between their means.

**Figure 4:**
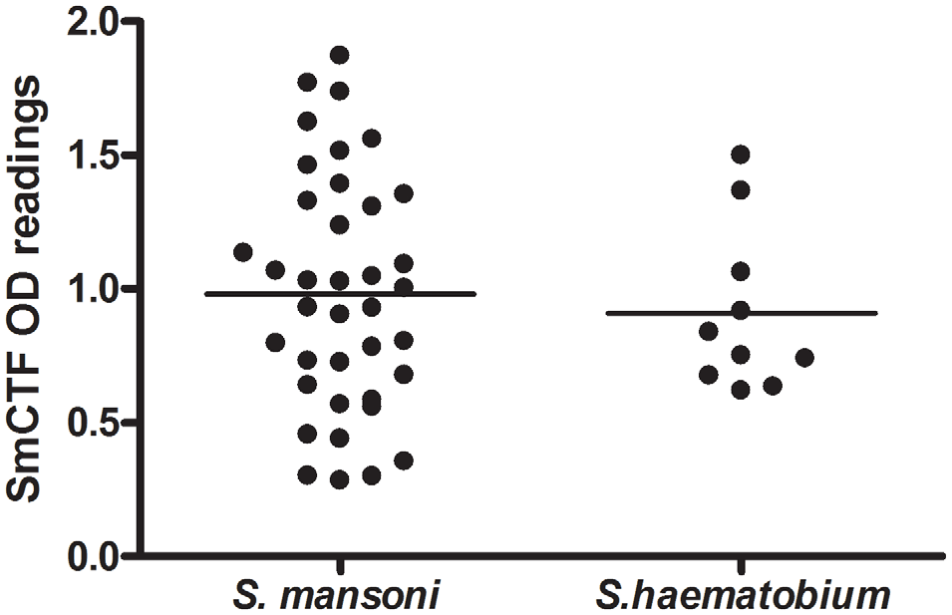
SPDRL: comparison of *S. mansoni*- and *S. haematobium*-infection sera in ELISA. Graph of the greater-than-cut-off OD450 results of *S. mansoni* infection sera in Figure 1 and *S. haematobium* infection sera in Figure 2 reacting against *S. mansoni* cercarial transformation fluid (SmCTF). Horizontal lines indicate the mean SmCTF OD450 values for each group of sera.

Figure 4 plots the individual results of the respective anti-SmCTF above cut-off value OD_450_ results from the *S. mansoni*-infection sera in Figure 1 and *S. haematobium*-infection sera in Figure 3. The mean of the latter values was marginally higher, but not significantly so.

### LSTM

**Figure 5:**
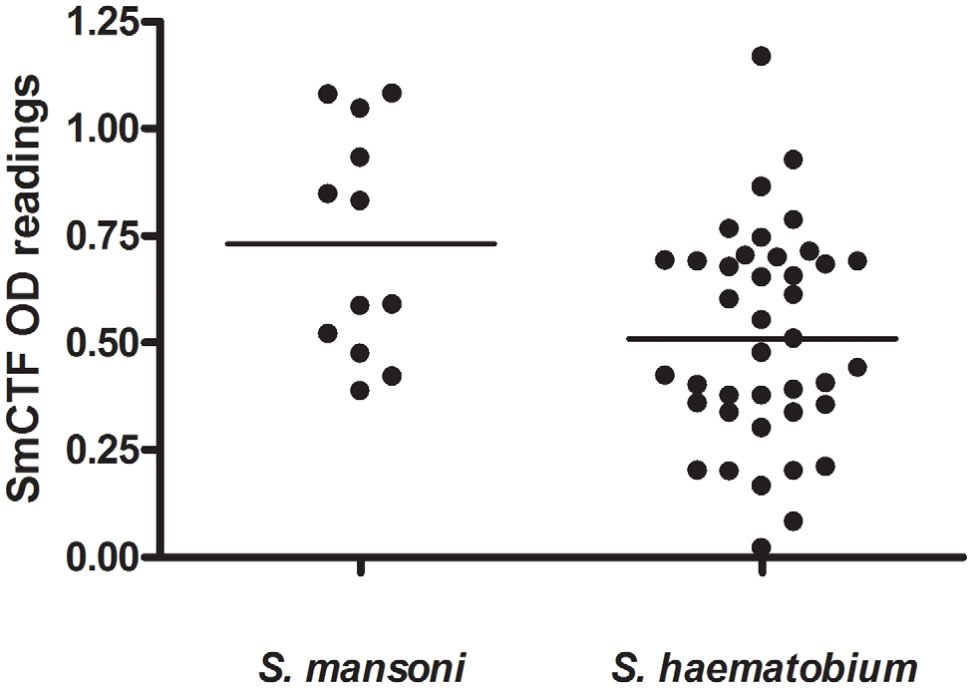
LSTM: comparison of *S. mansoni*- and *S. haematobium*-infection sera in ELISA. Reactivity of sera from *S. mansoni* and *S.haematobium* patients against *S. mansoni* cercarial transformation fluid (SmCTF). Lines indicate the mean SmCTF OD405 values for each group of sera.

In contrast to the results in Figure 4, LSTM found that 12 *S. mansoni* infection sera gave a higher mean OD_405_ reading with SmCTF than 40 *S. haematobium* infection sera (Figure 5), a difference that was significant (p<0.02).

**Figure 6:**
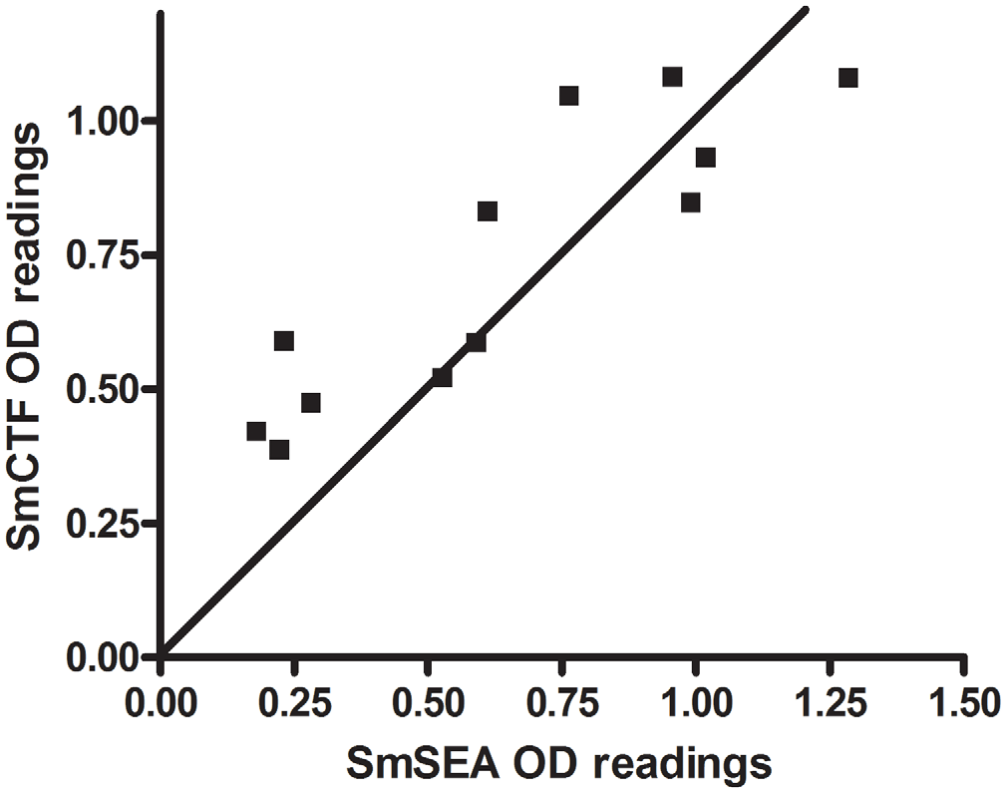
LSTM: correlation between *S. mansoni* cercarial transformation fluid (SmCTF) and soluble egg antigen (SmSEA) in ELISA. Results for sera from patients infected with *S. mansoni*.

**Figure 7:**
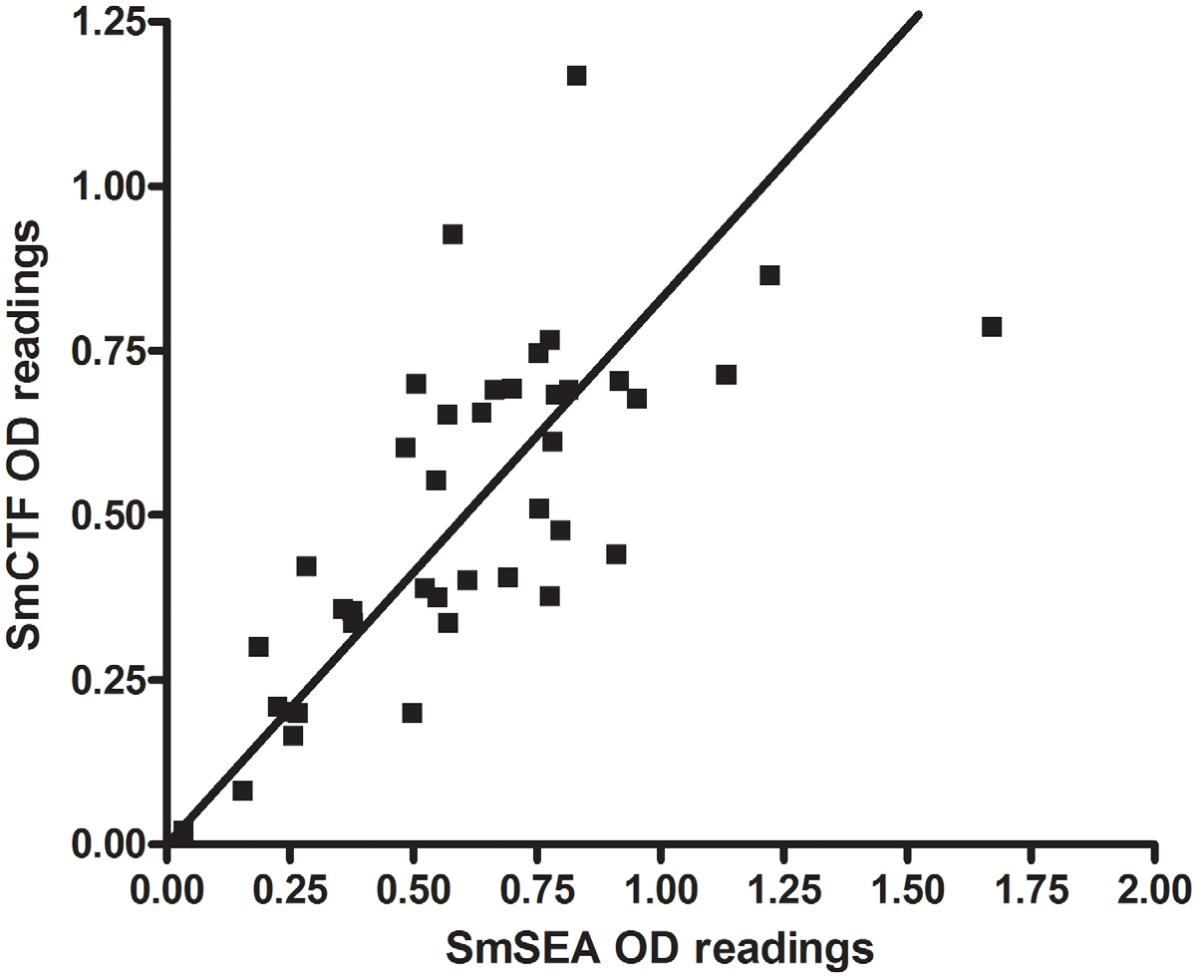
LSTM: correlation between *S. mansoni* cercarial transformation fluid (SmCTF) and soluble egg antigen (SmSEA) in ELISA. Results for sera from patients infected with *S. haematobium*.

**Figure 8:**
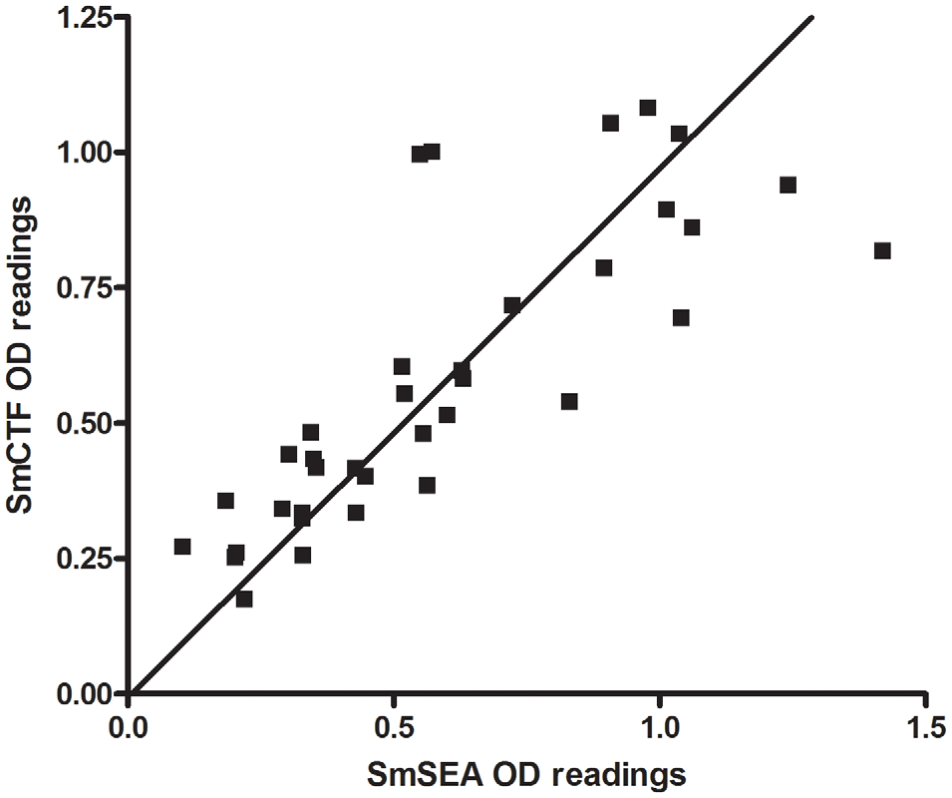
LSTM: correlation between *S. mansoni* cercarial transformation fluid (SmCTF) and soluble egg antigen (SmSEA) in ELISA. Results for sera from patients suspected to be infected with schistosomes, but not identifiable as either *S. mansoni* or *S. haematobium*.

When the reactions of individual sera against SmCTF were plotted against their respective reactions against SmSEA (Figure 6) the anti-SmCTF OD_405_ values of the majority of the *S. mansoni* infection sera appeared to be higher than their respective anti SmSEA OD_405_ values (although this difference is not significant), while the reactivities of the *S. haematobium* infection sera were relatively evenly distributed about the equivalence line (Figure 7). Similarly, sera from a group of subjects suspected to be infected with schistosomes, but not determinable as either *S. mansoni* or *S. haematobium*, reacted relatively similarly to both SmCTF and SmSEA in ELISA (Figure 8).

For the *S. mansoni* infection sera, there was 88.9% correlation between the anti-SmCTF OD_405_ values and the anti-SmSEA OD_405_ values (95% CI 64.4–96.9; p = 0.0001). For the *S. haematobium* infection sera, there was 77.4% correlation between the anti-SmCTF OD_405_ values and the anti-SmSEA OD_405_ values (95% CI 60.4–87.7; p<0.0001). For the suspected cases, there was 85.4% correlation (95% CI 73.4–92.2; p<0.0001).

Using either egg microscopy or the SEA-ELISA as the ‘gold-standard’ diagnostic test, the CTF-ELISA had a sensitivity of 100% to *S. mansoni* infections as there were no false-negative results. The SEA-ELISA gave one false-negative result with *S. mansoni* infection sera and therefore had a sensitivity of 91.6%.

Again using egg microscopy as the ‘gold-standard’, the CTF-ELISA had a sensitivity of 87.5% to *S. haematobium* infections with 5 false-negative results. Two of these were very close to the cut-off value of 0.200 (0.198 and 0.199), and if these are taken to be positive results sensitivity is increased to 92.5%. One of the remaining false-negatives was with serum from a patient with mainly calcified eggs in the urine; this serum sample also tested negative in the SEA-ELISA. The SEA-ELISA had a sensitivity of 92.5% with 3 false-negative results. One of these false negatives gave an OD reading that was close to the cut-off value (0.188). Using the SEA-ELISA as the gold-standard test, the CTF assay had a sensitivity of 91.9% or 97.3% if the two-values that were close to the cut-off are taken to be positive results.

All of the sera from cases of suspected schistosomiasis gave a positive result with at least one of the antigens in ELISA. Using SEA in ELISA as the gold standard, the CTF-ELISA had a sensitivity of 97% with only one false-negative result. There were two false-positive results, which could reflect the CTF being more sensitive than SEA.

### AMC

**Figure 9:**
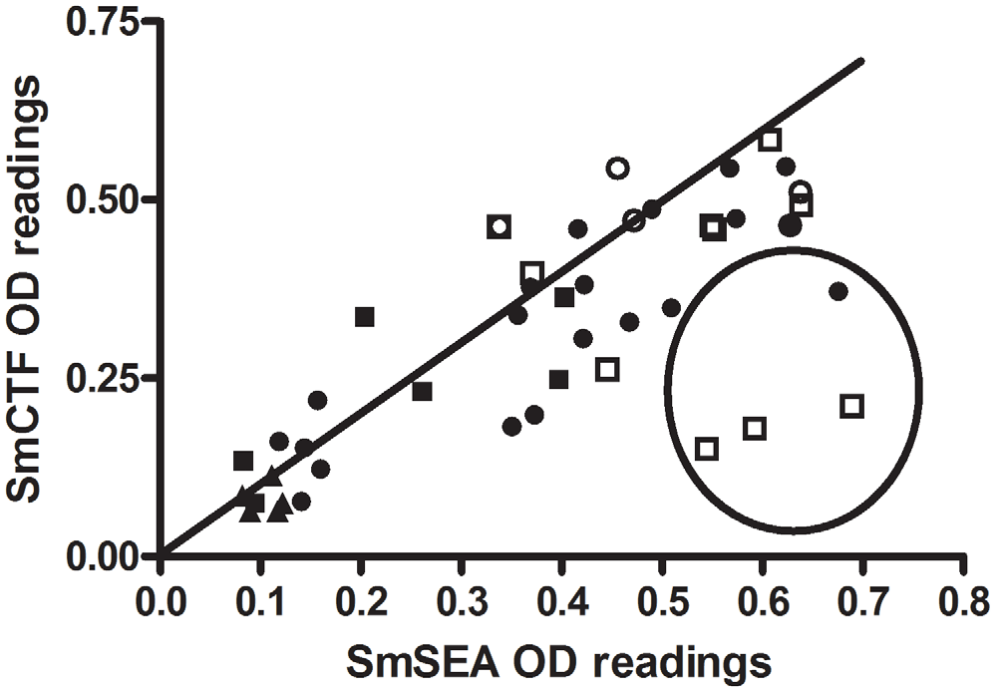
AMC: correlation between *S. mansoni* cercarial transformation fluid (SmCTF) and soluble egg antigen (SmSEA) in ELISA. Results for sera from a total of 46 subjects: 21 from patients with parasitologically-proven schistosome infections, 20 sera that were positive in routine serology and 5 negative controls. □ = sera from patients known to be *S. mansoni*-infected; ○ = sera from patients known to be *S. haematobium*-infected; ▪ = sera from patients with Katayama fever; • = sera from patients that tested positive for schistosomiasis in routine serology, but were egg-negative; ▴ = control negative sera from patients that tested negative in routine serology.

Figure 9 shows SmCTF OD_492_ values plotted against the respective SmSEA OD_492_ values of a total of 46 sera; 21 from patients with parasitologically-proven *Schistosoma* infection, 20 sera that were positive in routine serology and 5 negative controls. These differ somewhat from the results of the SPDRL and LSTM; in particular a few had high SmSEA ODs and low SmCTF ODs (see ringed points in figure 9). Three of these points are the results from patients with parasitologically-proven *S. mansoni* infection (two of which gave negative SmCTF-ELISA results); the other is not associated with any one infection species or status. Despite these outlying results, there was 72.45% correlation (95% CI 54.4–84.1; p<0.0001).

In contrast to the SPDRL's results, the majority of individual anti-SmSEA OD readings (mean = 0.534, SD = 0.114) are greater than the respective anti-SmCTF OD values (mean = 0.365, SD = 0.152) for *S. mansoni* infection sera, a difference which is significant (p = 0.0124). Also, in contrast to the results from the SPDRL and LSTM (figures 4 and 5), the AMC found that 5 *S. haematobium*-infection sera gave a higher mean OD reading with SmCTF (mean = 0.49, SD = 0.15) than 10 *S. mansoni* infection sera (mean = 0.36, SD = 0.04), a difference that was significant (p<0.05).

### NMU

Sera from 42 subjects living in the vicinity of Poyang Lake, Jiangxi Province, China, an area endemic for *S. japonicum*, and from 20 volunteers with no history of living in a schistosome endemic area were tested for reactivity in ELISA against 3 antigens: SmCTF, SmSEA and SjSEA, the last of these three being the antigen routinely used to detect anti-*S. japonicum* antibodies in this laboratory.

**Figure 10:**
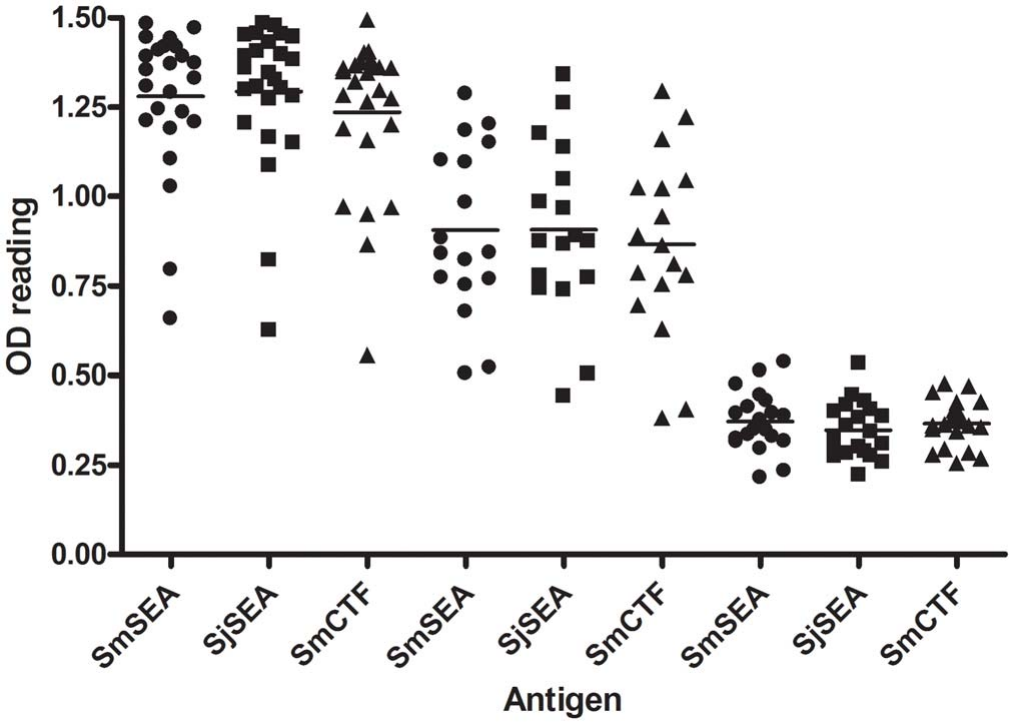
NMU: reactivity of sera from the Peoples Republic of China against *S. mansoni* SEA (SmSEA), *S. japonicum* SEA (SjSEA) and *S. mansoni* CTF (SmCTF) in ELISA. Twenty five sera were from *S. japonicum* egg-positive subjects and 17 from egg-negative subjects, both groups living in an area endemic for schistosomiasis japonicum, and 20 sera from negative controls living outwith an endemic area. Horizontal lines indicate the mean OD450 values for each antigen in each of the subject groups. • SmSEA ▪ SjSEA ▴ SmCTF.

The individual OD_450_ results for the 3 groups of sera against the 3 different antigens are shown in Figure 10. The mean reactivities of each of the 3 groups against each antigen are very similar. (Egg-positives: SmSEA mean = 1.28, SD = 0.20; SjSEA mean = 1.29, SD = 0.20; SmCTF mean = 1.24, SD = 0.22; egg negatives: SmSEA mean = 0.91, SD = 0.24; SjSEA mean = 0.91, SD = 0.24; SmCTF mean = 0.87, SD = 0.26; negative controls: SmSEA mean = 0.37, SD = 0.08; SjSEA mean = 0.35, SD = 0.08; SmCTF mean = 0.37, SD = 0.07). The 3 respective groups of sera gave very similar OD_450_ values irrespective of whether an egg antigen preparation from either *S. japonicum* (mean = 0.88, SD = 0.44) or *S. mansoni* (mean = 0.88, SD = 0.43), or the *S. mansoni* cercarial antigen preparation (mean = 0.85, SD = 0.42) was the target in ELISA. The correlation between the anti-SjSEA OD_450_ values and the anti-SmCTF OD_450_ values was 96.6% (95% CI 94.2–98.0; p<0.0001). There was 96.4% correlation between the anti-SmSEA OD_450_ values and the anti-SmCTF OD_450_ values (95% CI 94.0–97.9 (p<0.0001), and 98.5% correlation between the anti-SjSEA OD_450_ values and the anti-SmSEA OD_450_ values (95% CI 97.5–99.1; p<0.0001).

The OD_450_ values that discriminated between a +ve and −ve outcome for each serum in this experiment were calculated as the mean+2× SD of the uninfected control OD_450_ values of the uninfected sera. The cut-off values thus calculated were for: SmSEA = 0.539, SmCTF = 0.498 and SjSEA = 0.502. All three of the antigens used in ELISA gave positive results on the sera from egg-positive cases. Most of the sera from endemic egg-negative cases tested positive with all three antigens in ELISA, i.e. gave false-positive results compared to egg microscopy. All of the negative controls gave negative ELISA results with the three antigens, except for one which gave a false-positive result with SjSEA.

Using SjSEA in ELISA as the ‘gold standard’ for diagnosis of *S. japonicum* infection, SmCTF had 95.24% sensitivity and 100% specificity. The two ‘false-negatives’ seen were due to positive results with SjSEA on a negative control and an egg-negative case. SmSEA gave exactly the same positive and negative results as SmCTF, and so also had 95.24% sensitivity and 100% specificity compared to SjSEA. SmSEA and SmCTF had a positive predictive value (PPV) of 100% and a negative predictive value (NPV) of 90.9%.

Using egg microscopy as the gold standard, SmCTF had a sensitivity of 100% and a specificity of 62.5%. 15/17 of the endemic area egg-negative cases gave positive results with SmCTF in ELISA. There were no false-negatives. SmSEA had the same sensitivity and specificity values as SmCTF. SjSEA in ELISA compared with egg microscopy had a sensitivity of 100% and was 59.5% specific; i.e., less specific than SmCTF and SmSEA as there were 2 more false-positives using SjSEA. There were no false-negatives. SmSEA and SmCTF had a PPV of 62.5% and a NPV of 100%; SjSEA had a PPV of 59.5% and a NPV of 100%.

A flow-chart summarizing the results from the 5 laboratories is given in supporting information Figure S1.

## Discussion

The above results indicate that in three different laboratory settings the novel SmCTF antigen preparation is as effective in detecting anti-*S. mansoni*, anti-*S. haematobium* and anti-*S. japonicum* antibodies as SmSEA. The latter antigenic preparation, derived from *S. mansoni* eggs, has been routinely used to diagnose schistosome infections in the 3 European laboratories participating in this study, all of which have diagnostic service responsibilities.

There were some minor differences between the 3 European laboratories, perhaps a reflection of the way that each performs its ELISA reactions and incidentally an indication that there is as yet no standardized and widely used method for detecting anti-schistosome antibodies.

The results from the SPDRL show that for the suspected *S. mansoni*-infection sera the sensitivity of SmCTF relative to SmSEA is high with only one false-negative in 59 results. Specificity is not so high: 80% as a result of 5 false-positives, but this could be due to failure of the SmSEA to detect light infections [3]. It could however alternatively be due to sensitisation by cercarial antigens of bird and animal schistosome species, though an association between these and the White Nile in Uganda has seldom if ever been reported. Indeed, cross-reactivity between SmCTF and antibodies from cercarial dermatitis patients will need to be investigated before this assay is fully validated.

For the *S. haematobium*-infection sera examined in SPDRL SmCTF was 100% sensitive and 100% specific as there were no false-negatives or false-positives relative to SmSEA.

The AMC results differed from those of the other two European laboratories since those infection sera with higher SmSEA OD readings had lower SmCTF OD readings and three of them were parasitologically-proven *S. mansoni*-infection sera. Two of these gave false-negative results. The sample size here was, however, considerably smaller than that of the other two laboratories with only 10 *S. mansoni*-infection sera tested.

At the SPDRL, SmCTF gave higher OD readings with *S. mansoni*-infection sera than SmSEA (p<0.05), but this was not the case in the other two laboratories; the LSTM's results showed little distinction between SmCTF and SmSEA with regard to the OD values given by individual sera in ELISA, and at the AMC SmSEA gave higher OD readings than SmCTF with *S. mansoni* infection sera (p<0.02). Again there is the problem of a small sample size with the results from the AMC.

It is perhaps surprising that the *S. haematobium*-infection sera reacted well with the *S. mansoni* antigen preparations in all three laboratories, both cercarial-derived (SmCTF) and egg-derived (SmSEA). At the AMC they reacted better with SmCTF than the *S. mansoni*-infection sera (p<0.05), but at the LSTM *S. mansoni* sera reacted better than *S. haematobium* sera (p<0.02) and there was no significant difference between the two at the SPDRL.

It has long been known that anti-cercarial antibodies in human infection sera can be demonstrated, for example by means of the so-called cercarienhüllen reaction [33], but schistosome cercariae have not often been used to detect antibodies in immunodiagnosis of schistosomiasis, perhaps because of a relative lack of sensitivity and specificity [3], [34]. Higher anti-larval:anti-adult antibody levels may be useful in discriminating between acute and chronic infection [35]. Two more recent studies have indicated that the novel cercarial antigen SmCTF performs equivalently to SmSEA for the diagnosis of both *S. mansoni* and *S. haematobium* infections [27], [28].

It is suspected that the anti-schistosome antibodies that were detected by the SmCTF-ELISA had been induced by immunogens derived from eggs lodged in the tissues of infected humans (rather than by the schistosome cercariae or worms that caused the infection). Furthermore, the carbohydrate molecules derived from the cercarial glycocalyx and which become solubilised during the transformation of cercariae to schistosomula are suspected to be the principal antigenic moiety in SmCTF reactive here in ELISA. The serological cross-reactivity indicated by the present results, both between schistosome species and between antigens derived from schistosome cercarial and egg stages, is likely to be due to the presence of the same carbohydrate epitopes on the molecular constituents of eggs and larvae of the different species [22], [23], [36]–[38]. In view of the likely high dependence of the anti-cercarial antigen antibody reactivity being induced by egg antigens, it seems improbable that SmCTF will be of use in detecting pre-patent or single-sex worm-alone infections. It seems to us less likely that protein epitopes are involved to any significant extent in the antigen/antibody reactions described here because the reactivity of human infection sera against SmCTF in Western immunoblots is negated by prior treatment of the antigens with sodium metaperiodate solution (Francklow and Doenhoff, unpublished result). Furthermore, antibodies reactive against one of the principal protein constituents of SmCTF, the cercarial elastase, seem not to be present in human infection sera [39].

Many of the schistosome-infected subjects encountered by the three European laboratories involved in this study during the course of their diagnostic service responsibilities have light levels of infection which routine parasitological microscopy and antigen-detection methods often are too insensitive to detect. Despite their disadvantages, antibody-detection methods have therefore to be resorted to in the absence of any other useful test to detect infection directly. Because it is such a safe drug, praziquantel is then often automatically administered to those who are antibody-positive, despite a risk of some of the antibody-detection diagnostic test results being falsely-positive with regard to active infection.

Praziquantel has become the basis for control of endemic schistosomiasis and has begun to be used on a large scale in mass drug-administration programmes [40]–[43]. Microscopical parasitology, particularly the Kato-Katz method, has generally been used to estimate epidemiological parameters prior to commencement of these control programmes. If, however, control is successful the rate of schistosome egg excretion will decline in treated populations and Kato-Katz is likely to become ever less useful due to its relative insensitivity [3], [11], [17], [34]. In the absence of effective alternative methods to detect schistosome infections directly it is now being anticipated that antibody detection methods will become increasingly useful [17]. The importance of developing novel diagnostic tools appropriate to the changing requirements of control programmes is now becoming widely recognised, despite this research area often being thought of as less important than that of others, such as drug and vaccine development [11], [43], [44].

Stothard has recently evaluated the potential of diagnostic tests in improving control of schistosomiasis [45] and SmSEA in the ELISA format has been shown to hold promise as a diagnostic method for the monitoring of this disease in Zanzibar [46]. SEA-ELISA tests are the frequent method of choice in travellers' medicine clinics [18] and there is also a commercially-produced SmSEA-ELISA kit now available for use in the field [46]. Indeed, SEA-ELISA formats have previously given meaningful results in endemic areas [21], [47]. The production of egg antigens is however a fairly laborious and expensive process as the eggs have to be produced from infected animals. The use of SmCTF as a replacement for SmSEA would be a cost-effective option as SmCTF is harvested from infected snails, which are relatively easy to culture and maintain. This would also reduce the number of mice that are required, in comparison to those needed for the production of SmSEA. We estimate the overall animal-usage costs of producing SmCTF to be as much as 90% less than to produce an equivalent antigenic biomass from eggs.

As well as the expense of SmSEA, use of ELISA is also problematic outside the laboratory because of power requirements for blood centrifugation and electronic reading of ODs. Point-of-care (POC) or rapid diagnostic tests (RDTs) that are scalable and cost-effective for use in the developing world are becoming increasingly useful for the diagnosis of helminth infections [11]. RDTs that work on whole blood are likely to be useful for the implementation of control programmes in countries endemic for schistosomiasis, provided their application improves the efficiency of allocating praziquantel treatments and their costs can thus be off-set at least in part by a reduction in drug-wastage as a result of those who are antibody-negative being left untreated [45]. As the SmCTF antigen is cheap to produce and appears to be as good as SmSEA in these preliminary experiments and others [27], [28], a rapid test incorporating SmCTF to detect anti-schistosome antibodies, in a format usable with whole blood as well as serum, is being developed. It is hoped that it can be produced for sale at a cost of <US$1.00 which would make its use for control of schistosomiasis in endemic areas economically justified [45]. Of course such an assay would need to be evaluated in endemic areas prior to implementation.

There is currently a commercially available RDT on the market for diagnosis of both intestinal and urinary schistosomiasis by detection of circulating antigens (marketed by Rapid Medical Diagnostics, South Africa), but this test has recently been shown to be unsatisfactory for diagnosis of *S. haematobium* infections in Zanzibar [46], [48], despite being a reformulation of a test that encountered problems previously. The circulating cathodic antigen (CCA) urine-dipstick was however found to be an effective means of testing for intestinal schistosomiasis in shoreline communities of Lake Victoria [49].

Because the CCA-RDT has been marketed at US$2.3–2.8 per test [49], and more recently at $1.98 [50], an RDT using SmCTF to detect anti-schistosome antibodies would be a more cost-effective alternative, with great benefits to endemic areas, as many are resource-poor. As the results above indicate SmCTF is as good as SmSEA in detecting anti-schistosome antibodies in ELISA and thus decidedly a promising technique for the diagnosis and monitoring of schistosomiasis. Our results also show that SmCTF reacts well with both *S. mansoni* and *S. haematobium* infection sera, an advantage over the urine-CCA strips currently on the market.

Definitive diagnosis of schistosomiasis japonica is still very reliant on the demonstration of viable ova in faeces or other histological samples, despite the fact that parasitological techniques have now become relatively insensitive following widespread, repeated chemotherapy [51]. Immunodiagnostic technology was incorporated into the national control program in China in the 1980s as a way of improving identification of individuals as targets for treatment [52]. The Chinese national control program recommends the use of immunodiagnostic assays for the screening of populations in schistosome-endemic areas with a prevalence of less than 20% [53]. Immunodiagnosis is also used for preliminary screening in areas where the prevalence rate is less than 5% [52]. Among the techniques that have been successfully applied in the field is the ELISA incorporating *S. japonicum* soluble egg antigens (SjSEA) for detection of the anti-schistosome antibodies. The assay has high sensitivity and good specificity in diagnosis of *S. japonicum* infections [53]–[55], but the same problems as those with the SmSEA-ELISA are encountered: SjSEA is expensive to produce, there is the problem of requiring sera for ELISA and the ELISA format is generally not suitable for use outside of a designated laboratory. The results here show that SmCTF performs equivalently to SjSEA in ELISA with *S. japonicum* infection sera. This suggests not only that SmCTF could replace SjSEA in ELISA for diagnosis of *S. japonicum* infections, but also that an RDT incorporating SmCTF for diagnosis of *S. mansoni* and *S. haematobium* infections could have potential in the diagnosis of schistosomiasis japonica.

In conclusion, the SmCTF antigen appears to perform equivalently to SmSEA in ELISA with *S. mansoni* and *S. haematobium* infection sera, as well as with *S. japonicum* sera. SmCTF is more easily and cheaply produced than SmSEA, with the added advantage of a reduced number of laboratory animals required for antigen production. As well as replacing SmSEA in ELISA, the SmCTF could have potential in the development of an RDT that detects anti-schistosome antibodies in whole blood-usable format. If this RDT could be marketed at <US$1.00 per test its application in schistosomiasis control programmes in endemic areas would seemingly be justified. Development and evaluation of a RDT incorporating SmCTF as the antibody target is now underway.

## Supporting Information

Figure S1 **A flow-chart summarizing the number of sera tested for reactivity against SmSEA and SmCTF in ELISA in the 5 laboratories, and the outcome in terms of number of sera giving positive and negative reactions.**
(DOCX)

Checklist S1 **A STARD (STAndards for the Reporting of Diagnostic accuracy studies) checklist** **[56]**, **[57]** **.**
(DOC)
