# Supplementary material for: Comparison of Schistosoma mansoni Soluble Cercarial Antigens and Soluble Egg Antigens for Serodiagnosing Schistosome Infections
Source: PLoS Negl Trop Dis. 2012 Sep 13;6(9):e1815. doi: 10.1371/journal.pntd.0001815 (PMC3441401; doi:10.1371/journal.pntd.0001815)
Supplement: Figure S1 — A flow-chart summarizing the number of sera tested for reactivity against SmSEA and SmCTF in ELISA in the 5 laboratories, and the outcome in terms of number of sera giving positive and negative reactions. (DOCX) [file pntd.0001815.s001.docx]

**STARD Flowchart**

Number of patient sera used in total across the four laboratories = 241

Number also positive by the SmCTF-ELISA = 187

Number positive by the SmCTF-ELISA = 10

Number also negative by the SmCTF-ELISA = 36

Number negative by the SmCTF-ELISA = 8

Number of patients negative by the SmSEA-ELISA reference test = 46

Number of patients positive by the SmSEA-ELISA reference test = 195

Number of sera tested by the SmSEA-ELISA reference test = 241
